# Anagliptin prevented interleukin 1β (IL-1β)-induced cellular senescence in vascular smooth muscle cells through increasing the expression of sirtuin1 (SIRT1)

**DOI:** 10.1080/21655979.2021.1948289

**Published:** 2021-07-21

**Authors:** Juan Zhao, Xinrong He, Mei Zuo, Xinguo Li, Zhiming Sun

**Affiliations:** a Department of Cardiovascular Medicine, Xianyang Hospital of Yan’an University, Xianyang, Shaanxi, China; b Department of Cardiology, The Fourth People’s Hospital of Shaanxi, Xi’an, Shaanxi, China

**Keywords:** Anagliptin, vascular smooth muscle cells, cell senescence, sirt1, il-1β, atherosclerosis

## Abstract

Vascular smooth muscle cell senescence plays a pivotal role in the pathogenesis of atherosclerosis. Anagliptin is a novel dipeptidyl peptidase-4 (DPP-4) inhibitor for the treatment of hyperglycemia. Recent progress indicates that DPP-4 inhibitors show a wide range of cardiovascular benefits. We hypothesize that Anagliptin plays a role in vascular smooth muscle cell senescence and this may imply its modulation of atherosclerosis. Here, the beneficial effect of Anagliptin against interleukin 1β (IL-1β)-induced cell senescence in vascular smooth muscle cells was studied to learn the promising therapeutic capacity of Anagliptin on atherosclerosis. Firstly, we found that Anagliptin treatment ameliorated the elevated secretions of tumor necrosis factor-α (TNF-α), interleukin 6 (IL-6), and macrophage chemoattractant protein-1 (MCP-1). Secondly, our findings indicate that exposure to IL-1β reduced telomerase activity from 26.7 IU/L to 15.8 IU/L, which was increased to 20.3 and 24.6 IU/L by 2.5 and 5 μM Anagliptin, respectively. In contrast, IL-1β stimulation increased senescence- associated β-galactosidase (SA-β-gal) staining to 3.1- fold compared to the control group, it was then reduced to 2.3- and 1.6- fold by Anagliptin dose-dependently. Thirdly, Anagliptin dramatically reversed the upregulated p16, p21, and downregulated sirtuin1 (SIRT1) in IL-1β-treated vascular smooth muscle cells. Lastly, the protective effect of Anagliptin against cellular senescence in vascular smooth muscle cells was abolished by silencing of SIRT1. In conclusion, Anagliptin protects vascular smooth muscle cells from cytokine-induced senescence, and the action of Anagliptin in vascular smooth muscle cells requires SIRT1 expression.

## Graphical Abstract

**Figure:**
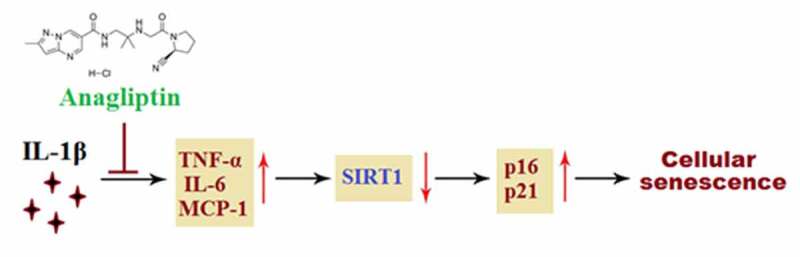

## Introduction

Cell senescence is defined as the permanently irreversible stagnation of the cell cycle with the specific characteristics of cell morphology, gene expression, and cellular metabolism, which is different from cell arrest and cell apoptosis [1]. As the investigations on cell senescence further develop, several characteristics have been proven to be useful in determining the state of cell senescence. Firstly, the morphology of cells under the state of cell senescence tends to be flat and wide. In addition, a series of changes to gene expression, chromatin structure, cellular function, and cell phenotypes, such as the activation of SA-β-Gal, the up-regulation of cell cycle regulators, the shortening of the telomere, DNA damage, and the formation of the senescence-associated secretory phenotype (SASP) and senescence-associated heterochromatin foci (SAHF) [2]. It is reported that the cell senescence of vascular smooth muscle cells, is the early symbol of atherosclerosis, which is the basis of multiple types of cardiovascular diseases [3]. As the pathogenesis of atherosclerosis develops, vascular smooth muscle cells migrate to the tunica intima and accumulate around necrotic foam cells to form fibrous caps, which further contributes to the development of atherosclerotic plaques [4,5]. Inflammatory factors, such as IL-1β, are known to induce the state of cell senescence in cells [6,7]. Oxidative stress-induced premature senescence of vascular smooth muscle cells (VSMCs) has been reported in both *in vitro* and *in vivo* experiments [8]. VSMC senescence is associated with the instability of plaque formation and repair after rupture, and the understanding of VMSC senescence may provide evidence of targeted treatment in atherosclerosis [9]. Several cell cycle regulators such as p53, p21, and p16 are essential landmarks of senescence [10]. P21 is the downstream target gene of p53 and can be induced by it. In addition, p21 is an inhibitor of the cell cycle and arrests it at the G1 phase by inhibiting cyclin E [11]. The transformation from the G1 phase to the S phase is regarded as one of the characteristics of cell senescence [12]. As an inhibitor of cyclin-dependent kinases (CDK), p16 suppresses the activity of CDK4/6 by binding with cyclin D, further blocking the progression of the cell cycle [13]. Recently, it has been reported that SIRT1 could inhibit the transcription and activation of downstream genes of p53 by directly inactivating p53, indicating a potential regulatory effect of SIRT1 on cell senescence [14]. In addition, SIRT1 is reported to exert anti-oxidative and anti-apoptotic properties, it is significantly downregulated in atherosclerotic tissues and its expression is negatively related to the level of total cholesterol and low-density lipoprotein [15]. Therefore, targeting cell senescence of vascular smooth muscle cells has become an important strategy for the treatment of atherosclerosis.

**Figure 1.**
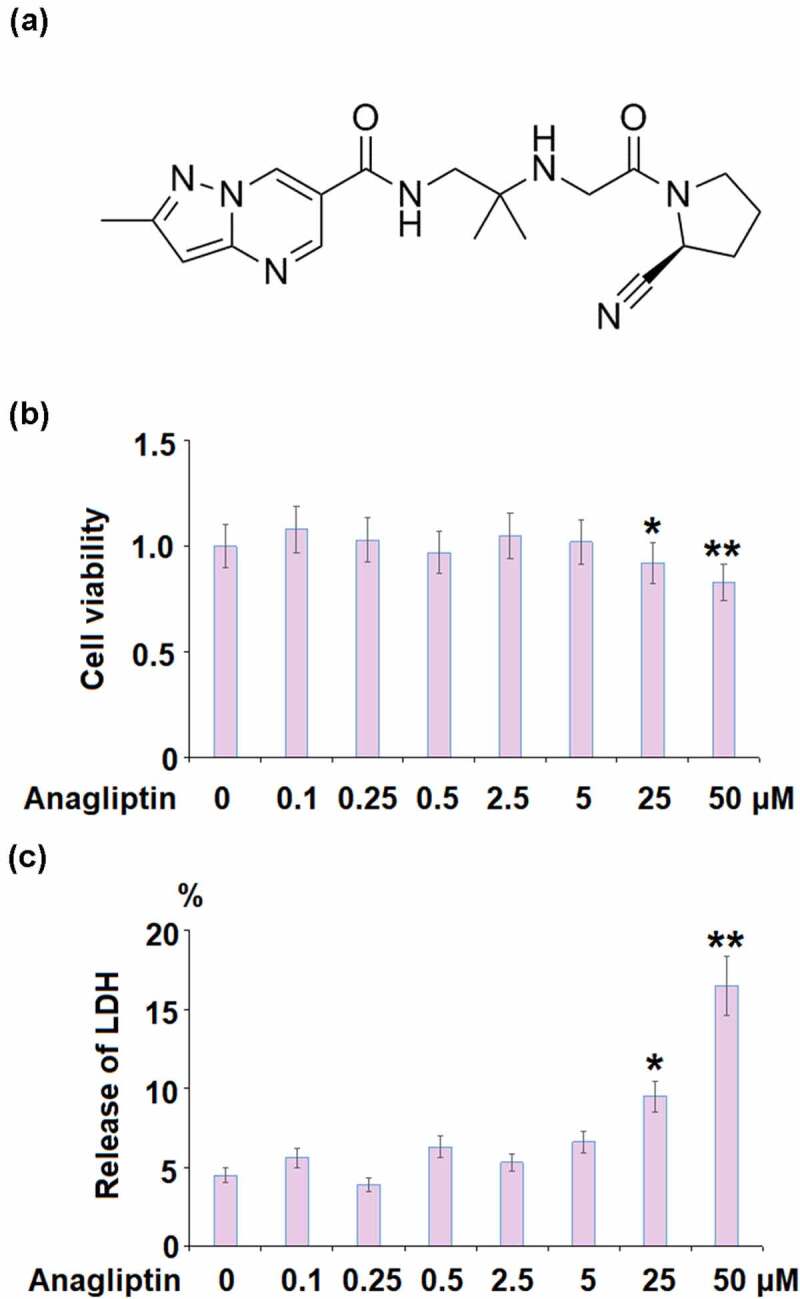
The effects of Anagliptin on cell viability of vascular smooth muscle cells. Cells were stimulated with Anagliptin at the concentrations of 0.1, 0.25, 0.5, 2.5, 5, 25, and 50 μM for 24 hours. (a). Molecular structure of Anagliptin; (b). Cell viability; (c). Release of LDH (*, **, P < 0.05, 0.01 vs. vehicle group)

Anagliptin (Figure 1a) is a novel compelling and specific inhibitor of dipeptidyl peptidase-4 (DPP-4) first approved for hyperglycemia therapy in Japan in 2012 [16]. Results of clinical trials revealed that Anagliptin exerted promising anti-diabetic effects, which could be enhanced by combination with acarbose [17,18]. Recently, potential therapeutic effects of Anagliptin against atherosclerosis have been reported. Hirano [19] claimed that in cholesterol-fed rabbits, the macrophage infiltration, and atherosclerosis in aortic and coronary arteries were significantly ameliorated by treatment with Anagliptin. Furthermore, Ervinna reported that in apoE-deficient mice, the proliferation of vascular smooth muscles and monocyte inflammation were both significantly suppressed and atherosclerosis was greatly alleviated by the administration of Anagliptin [20]. In addition, Anagliptin is reported to activate SIRT1 to ameliorate high glucose-induced endothelial dysfunction [21]. However, it’s unknown whether Anagliptin exerts a beneficial action against cellular senescence. Here, the protective effect of Anagliptin on IL-1β-induced cellular senescence in vascular smooth muscle cells was evaluated to judge whether Anagliptin possesses promising potential in the management of atherosclerosis.

## Materials and methods

### Cell culture and treatments

This study was performed in accordance with the principles of the World Medical Association Declaration of Helsinki for medical research using human-derived cells. Experimental protocols were approved by the Ethical Committee of Yan’an University. Human vascular smooth muscle cells (hVSMCs) were obtained from Guandao Biological Engineering (Shanghai, China) and cultured in a Dulbecco’s Modified Eagle Medium (DMEM) supplied with 10% fetal bovine serum (FBS) in 5% CO_2_ at 37°C. The treatment reagent IL-1β was purchased from R&D Systems (#201-LB), and Anagliptin was obtained from Sigma-Aldrich (#4943). To test the cytotoxicity of Anagliptin, hVSMCs were treated with a series of Anagliptin ranging from 0.1 to 50 μM. For all other experiments, cells were challenged with IL-1β (10 ng/mL) [22] or Anagliptin (2.5, and 5 μM) [20] for 24 hours. The cell treatment conditions were based on previous publications.

### Cell viability

Cells were plated in a 96-well plate (5000 cells per well). After necessary treatment, CCK-8 solution was added to the cells and incubated for 3 hours at 37°C. Lastly, optical density (OD) was determined using 450 nM.

### Release of lactate dehydrogenase (LDH)

The cytotoxicity was further confirmed by detecting the release of LDH using a commercial kit (#ab65391, Abcam, USA). Briefly, the cells were incubated with 1% Triton X-100 for 45 minutes, followed incubation of the samples in the dark in the cultural system containing lactate, NAD^+^, diaphorase, 0.004% BSA, 0.15% sucrose, and INT. After incubation for 30 minutes, OD at 490 nm was measured after eliminating the reaction.

### Real-time PCR analysis

In brief, an RNA isolation kit (Takara, Tokyo, Japan) was applied to isolate total RNAs from hVSMCs, followed by being transcribed into cDNA according to the instruction of the qRT-PCR mRNA Detection Kit (Takara, Tokyo, Japan). Subsequently, the Lightcycler 480 Detection System (Roche, Basel, Switzerland) was used to perform real-time PCR, with GAPDH taken as the internal control for normalization. The circle times were recorded for the calculation of target genes according to the 2^−ΔΔCt^ method. The following primers were used:

*MCP-1 (F: 5ʹ-AGAATCACCAGCAGCAAGTGTCC-3ʹ, R:5ʹ-TCCTGAACCCACTTCTGCTTGG-3ʹ); IL-6 (F: 5ʹ-TGGTCTTTTGGAGTTTGAGGTA −3ʹ, R: 5ʹ- AGGTTTCTGACCAGAAGAAGGA-3ʹ); TNF-α (5ʹ- CTCTTCTGCCTGCTGCACTTTG-3ʹ, 5ʹ- ATGGGCTACAGGCTTGTCACTC-3ʹ); p16 (5ʹ-CCCAACGCACCGAATAGTTA-3′, 5′-ACCAGCGTGTCCAGGAAG-3′), p21 (5ʹ- TGGAGACTCTCAGGGTCGAAA-3ʹ, 5ʹ-GGCGTTTGGAGTGGTAGAAATC-3ʹ), SIRT1 (F: 5ʹ -TCAGTGTCATGGTTCCTTTG-3ʹ, R: 5ʹ-AATCTGCTCCTTTGCCACTCT-3ʹ), GAPDH (5ʹ- GAACATCATCCCTGCCTCTACT −3ʹ, 5ʹ- GTCTACATGGCAACTGTGAGGA −3ʹ).*

### Western blot analysis

The total proteins were isolated from the treated hVSMCs using the lysis buffer, and quantified using the bicinchoninic acid (BCA) kit (Beyotime, Shanghai, China). Subsequently, the proteins were run on 10% sodium dodecyl sulfate polyacrylamide gel electrophoresis (SDS-PAGE) and further transferred to the polyvinylidene fluoride (PVDF) membrane (Bio-Rad, USA). After 3 washes with PBST buffer, the membrane was incubated with the primary antibody against p16, p21, SIRT1, and β-actin overnight in a cold room and incubated with HRP-conjugated secondary antibody (1:2000, CST, Boston, USA) at room temperature for 1.5 hours. Lastly, the membranes were exposed to an ECL detection system (CST, Boston, USA).

### Enzyme-linked immunosorbent assay (ELISA) assay

The concentrations of TNF-α, IL-6, and MCP-1 in the supernatant of treated hVSMCs were detected using the ELISA commercial kits (R&D Systems Europe, Abington, UK). The following Quantikine ELISA kits were purchased: IL-6 (#D6050), TNF-α (#PDTA00D), and MCP-1(#DCP00). TNF-α, IL-6, and MCP-1 were measured as per

the detailed procedure described previously [23].

### Telomerase activity

The CHAPS buffer was used to lyse the treated hVSMCs, followed by centrifugation at 15,000 × g for 30 minutes and quantifying the concentration of total proteins using the BCA kit (Beyotime, Shanghai, China). The TeloTAGGG Telomerase PCR ELISA Plus Kit (Roche, Basel, Switzerland) was used to measure the telomerase activity. The TRAP-PCR reaction system contained 0.5 mg of each sample and 10 pM of each primer, followed by quantification using RT-PCR assay.

### Senescence- associated β-galactosidase (SA-β-gal) staining

The Senescence Detection kit (Beyotime, Shanghai, China) was used. Briefly, cells were washed with PBS buffer and fixed using 2% paraformaldehyde for 5 min, followed by washing and incubated at 37°C in the absence of CO_2_ in fresh SA-β-gal staining solution. Lastly, the percentage of SA-β-gal positive blue cells was counted after being photographed.

### Statistical analysis

Analysis of Variance (ANOVA) with Tukey’s Multiple Comparison post-hoc test was used to compare the difference among groups. P < 0.05 was considered statistically significant.

## Results

We aimed to investigate whether Anagliptin possesses a protective effect against IL-1β- induced insults in VMSCs. We demonstrated that Anagliptin prevented cellular senescence by reducing SA-β-gal but increasing telomerase activity in IL-1β- challenged VMSCs. Mechanistically, we found that this effect was mediated by SIRT1.

### The effects of Anagliptin on cell viability of vascular smooth muscle cells

To test the cellular response to Anagliptin in IL-1β treated VMSCs, we performed the dose-response experiment. 10 ng/ml of IL-1β was used to treat VMSCs for 24 hours as described before [22]. The concentration of Anagliptin was based on previous report ^23^ and our test results. To test the cytotoxicity, cells were stimulated with Anagliptin at the concentrations of 0.1, 0.25, 0.5, 2.5, 5, 25, and 50 μM for 24 hours. No obvious difference was observed in the cell viability when Anagliptin was lower than 5 μM (Figure 1b). However, the cell viability decreased greatly as the concentration of Anagliptin was elevated to 25, and 50 μM. Similarly, the release of LDH was kept around 5% when Anagliptin was lower than 5 μM but was promoted to 9.5% and 16.5% by the introduction of 25, and 50 μM Anagliptin, respectively. Therefore, 2.5, and 5 μM were used as the incubation concentrations of Anagliptin in the subsequent experiments.

### Anagliptin reduced IL-1β-induced expression of pro-inflammatory cytokines in vascular smooth muscle cells

**Figure 2.**
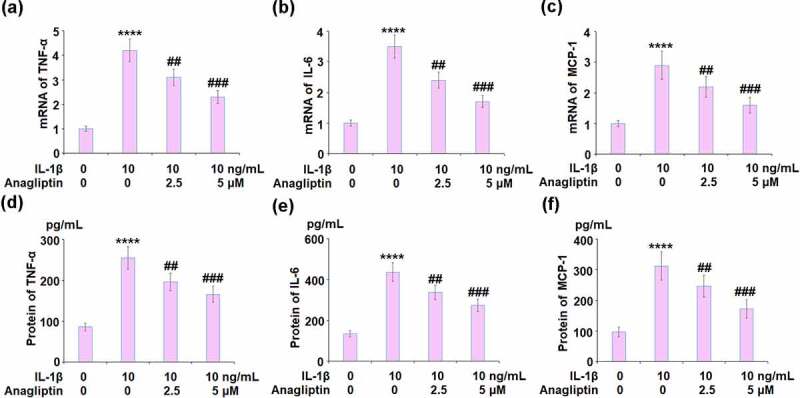
Anagliptin reduced IL-1β- induced pro-inflammatory cytokines. (a-c). mRNA of TNF-α, IL-6, and MCP-1; (d-f). Secretions of TNF-α, IL-6, and MCP-1 (****, P < 0.0001 vs. vehicle group, ##, ###, P < 0.1, 0.001 vs. IL-1β group)

The expressions of TNF-α, IL-6, and MCP-1 were remarkably increased by IL-1β and then suppressed by the introduction of Anagliptin (Figure 2a-c). We further detected the protein concentrations of TNF-α, IL-6, and MCP-1. The secretion of TNF-α was increased from 86.3 to 255.4 pg/mL by IL-1β then inhibited to 196.7 and 166.2 pg/mL by 2.5 and 5 μM Anagliptin, respectively. In addition, the secretions of IL-6 (Figure 2e) released in the control, IL-1β, 2.5 μM Anagliptin, and 5 μM Anagliptin groups were 135.1, 436.9, 337.5, and 273.8 pg/mL respectively. Lastly, the secretion of MCP-1 was elevated from 97.3 to 312.8 pg/mL by IL-1β but reduced to 246.7 and 172.5 pg/mL by the introduction of 2.5, and 5 μM Anagliptin, respectively.

### Anagliptin prevented IL-1β-induced cellular senescence

**Figure 3.**
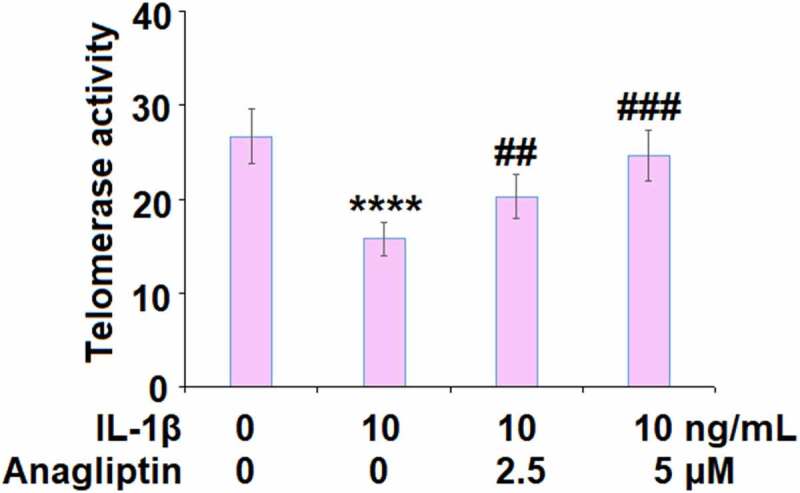
Anagliptin restored IL-1β-induced reduction of telomerase activity. Telomerase activity was measured using a commercial kit (****, P < 0.0001 vs. vehicle, ##, ###, P < 0.1, 0.001 vs. IL-1β)

**Figure 4.**
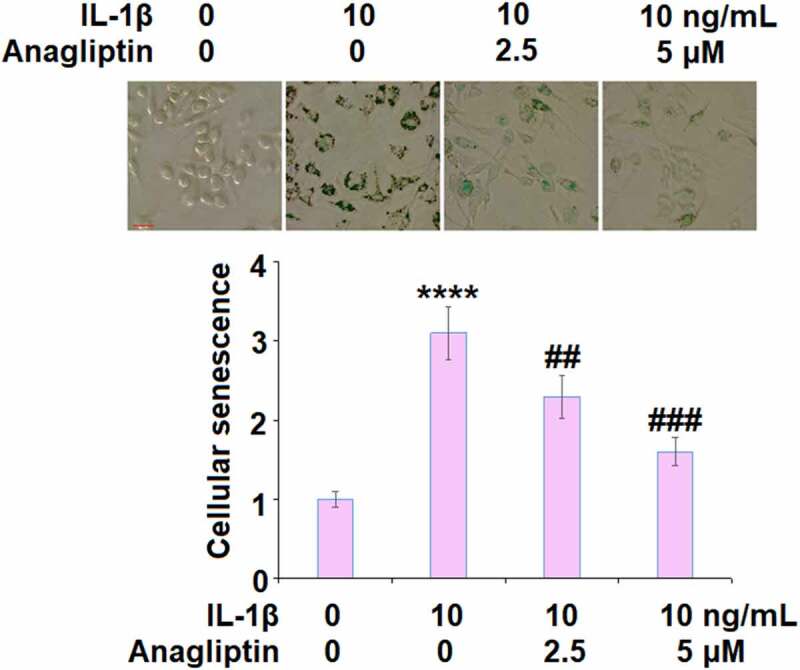
Anagliptin prevented IL-1β-induced cellular senescence. Cellular senescence was assayed using SA-β-gal staining. Scale bar, 100 μM (****, P < 0.0001 vs. vehicle, ##, ###, P < 0.1, 0.001 vs. IL-1β)

The telomerase activity was reduced from 26.7 IU/L to 15.8 IU/L by IL-1β but increased to 20.3 and 24.6 IU/L by 2.5, and 5.0 μM Anagliptin (Figure 3), respectively, indicating that Anagliptin remarkably attenuated the cellular senescence in vascular smooth muscle cells caused by IL-1β. We confirmed the effect of Anagliptin on cell senescence by performing the SA-β-gal staining assay. As expected, the activity of SA-β-gal was elevated by IL-1β but suppressed by the introduction of Anagliptin (Figure 4), confirming the beneficial effect of Anagliptin on IL-1β-induced cell senescence in vascular smooth muscle cells.

### Anagliptin reduced the expressions of p16 and p21

**Figure 5.**
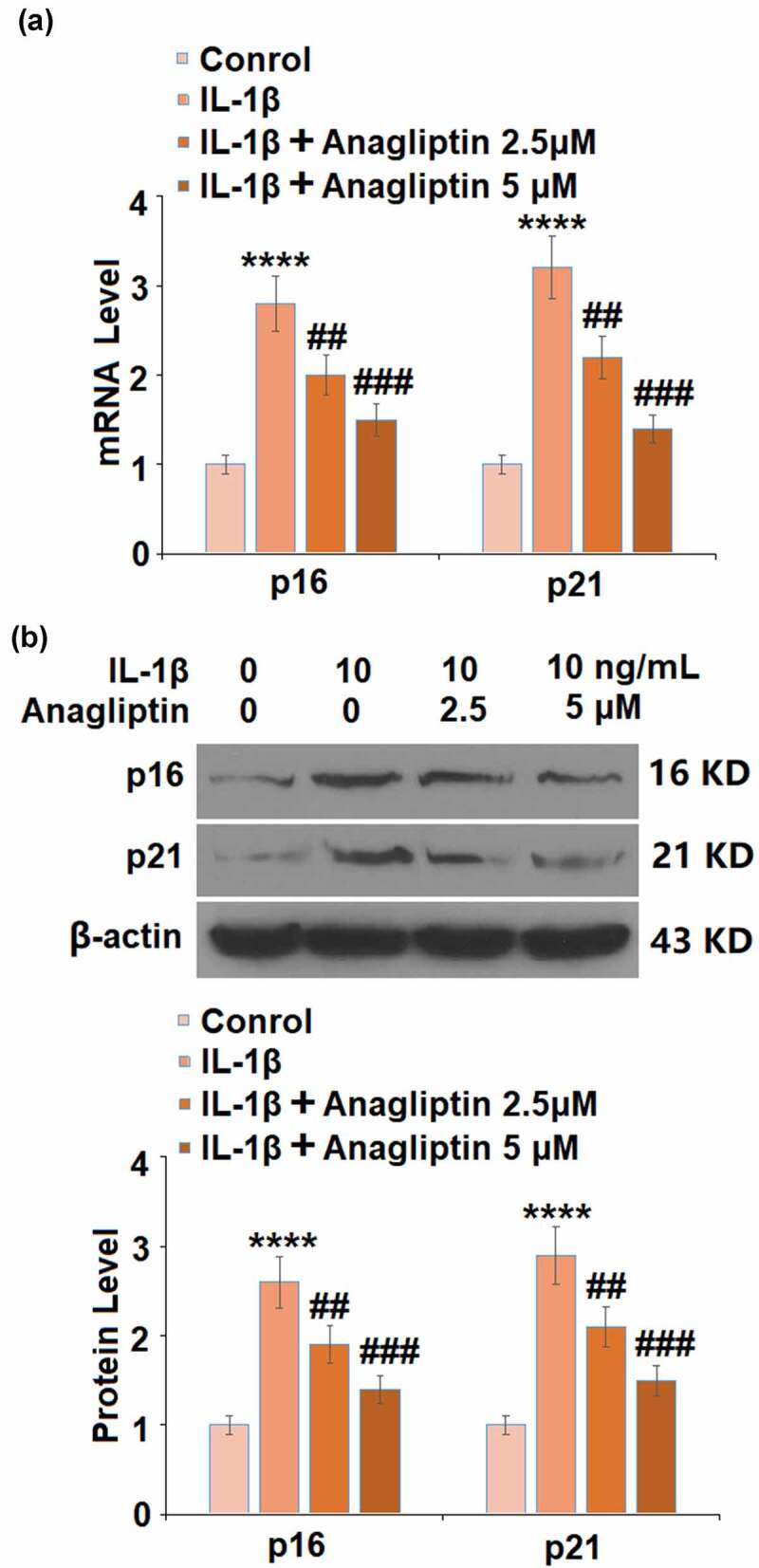
Anagliptin prevented IL-1β-induced p16 and p21. (a). mRNA of p16 and p21; (b). Protein of p16 and p21 (****, P < 0.0001 vs. vehicle group, ##, ###, P < 0.1, 0.001 vs. IL-1β group)

**Figure 6.**
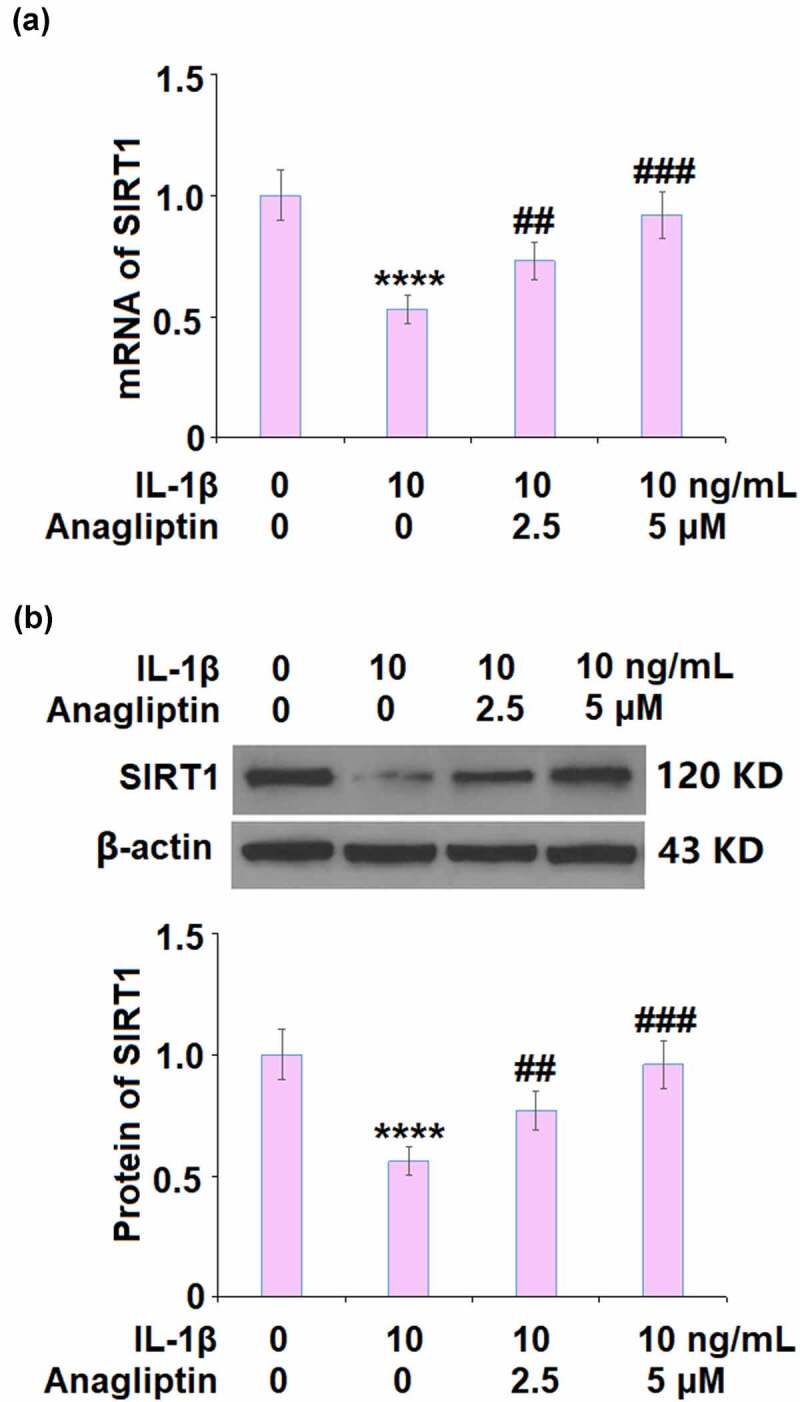
Anagliptin restored IL-1β-induced expression of SIRT1. (a). mRNA of SIRT1; (b). Protein of SIRT1 (****, P < 0.0001 vs. vehicle, ##, ###, P < 0.1, 0.001 vs. IL-1β)

To explore the potential mechanism of Anagliptin in vascular smooth cells, the expression levels of cell senescence-related proteins were determined. The expressions of p16 and p21 were markedly increased by IL-1β but greatly inhibited by Anagliptin (Figure 5). In addition, the downregulated SIRT1 (Figure 6) in IL-1β-treated vascular smooth muscle cells was upregulated by the introduction of Anagliptin.

### Silencing of SIRT1 abolished the beneficial effects of Anagliptin against cellular senescence in vascular smooth muscle cells

**Figure 7.**
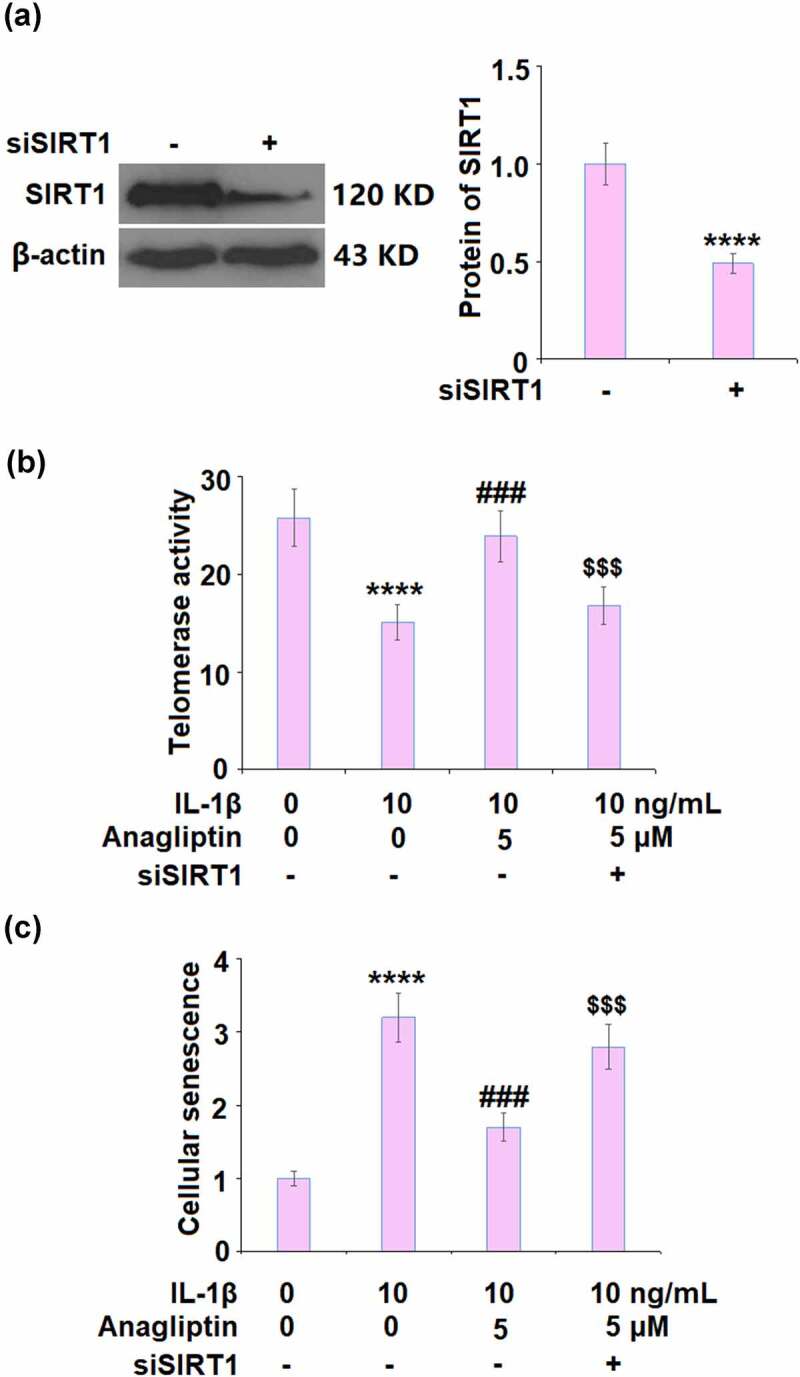
Silencing of SIRT1 abolished the protective effects of Anagliptin against IL-1β- induced cellular senescence. (a). Successful knockdown of SIRT1; (b). Telomerase activity; (c). Cellular senescence (****, P < 0.0001 vs. vehicle, ###, P < 0.001 vs. IL-1β; $$$, P < 0.001 vs. IL-1β+ Anagliptin group)

To further verify that the effects of Anagliptin on cell senescence were related to the upregulation of SIRT1, the expression of SIRT1 was silenced by transfection with SIRT1 siRNA. SIRT1 was obviously declined in siRNA-treated cells (Figure 7a), indicating that the knockdown of SIRT1 in vascular smooth muscle cells was successfully achieved. Interestingly, telomerase activity was reduced from 25.8 IU/L to 15.1 IU/L by IL-1β, and then increased to 23.9 IU/L by the treatment with 5 μM Anagliptin (Figure 7b). In Anagliptin-treated SIRT1 knockdown vascular smooth muscle cells, the telomerase activity was further decreased to 16.8 IU/L. In addition, the elevated activity of SA-β-gal (Figure 7c) induced by IL-1β was significantly suppressed by Anagliptin, reversed by the knockdown of SIRT1. These data indicate that Anagliptin might exert beneficial actions against IL-1β-induced cell senescence through up-regulation of SIRT1.

## Discussion

Telomere length is negatively related to the risk of cardiovascular diseases [24]. Compared to normal vascular wall cells and circulating endothelial progenitor cells [25], more significant shortened telomeres are observed in vascular smooth muscle cells [24] and endothelial cells [26] in atherosclerotic plaque. In addition to physiological senescence-related biological changes, the cells in the premature senescence state are accompanied by elevated expressions of inflammatory factors, further aggravating the damage to the tissues [27]. In this study, the *in vitro* cell senescence model was constructed on vascular smooth muscle cells by incubating the cells with IL-1β, which was verified by the decreased telomerase activity, elevated SA-β-gal, and increased secretion of inflammatory factors. By introducing optimized dosages of Anagliptin, the state of cell senescence and excessively released inflammatory factors in vascular smooth muscle cells were significantly reversed, indicating that Anagliptin might exert a promising anti-senescence effect against IL-1β-induced vascular smooth muscle cells.

P16 is encoded by INK4a/ARF located on 9p21, the N-terminal of which has the homologous structure to cyclin D. P16 impacts the phosphorylation of CDK4/6 retinoblastoma protein and the activation of downstream transcriptional factor E2F by competitively binding with cyclin D, arresting the cell cycle at the G1 phase [28]. Recently, p16 is reported to inhibit the cell cycle by inducing the degradation of the phosphorylated CDK4/6 retinoblastoma protein [29]. P53 is a stress protein and is rapidly upregulated when the cells are under stimulation, suppressing the activity of cyclinA/E-CDK2, inhibiting the phosphorylation of CDK4/6 retinoblastoma protein, and blocking the progression of the cell cycle by activating p21 [30]. Han reported that p16 could enhance the stability of p21 and that p16 could be activated by the interaction between p21 and Sp1, which contribute to the inhibition of the cell cycle synergistically [31]. Here, by the stimulation, the cell senescence was induced in vascular smooth muscle cells, accompanied by the upregulation of p16 and p21. After the treatment with Anagliptin, the expressions of p16 and p21 were suppressed significantly, indicating that Anagliptin might exert its anti-senescence property by inhibiting the p16 and p21 pathways. In our future work, the expressions of more related proteins, such as p53, E2F, and p-CDK4/6 retinoblastoma protein, will be detected to further confirm the effects of Anagliptin on the p16 and p21 pathways.

SIRT1 plays an important role in gene transcription, cell senescence, and energic metabolism by exerting a deacetylation function on such proteins as H1, H3, H4, p53, and forkhead box protein O (FOXO) [32]. Xia [33] reported that the activation of SIRT1 could suppress the expression of p16. In addition, the activation of the p53/p21 signaling pathway could be inhibited by SIRT1 by inducing the deacetylation on p21 or p53 [34,35]. We reported that the downregulated expression level of SIRT1 in IL-1β- challenged vascular smooth muscle cells was significantly reversed by Anagliptin, indicating that Anagliptin might regulate the p16 and p21 signaling pathway by activating SIRT1. We further confirmed the mechanism by establishing SIRT1-knockdown vascular smooth muscle cells. In our future work, more detailed investigations, such as detecting the deacetylation level of p21 and p53, will be conducted to better understand the regulatory effects of Anagliptin on SIRT1 and its downstream pathway.

The senescence of VSMCs accompanied by the gained senescence-associated secretory phenotype (SASP) could lead to vascular inflammation, hence the loss of vascular function [9]. Our data suggest Anagliptin effectively suppressed the production of key pro-inflammatory cytokines (TNF-α, IL-6, and MCP-1) and the expressions of senescence pathways (p53/p21 and p16), implying its multiple regulation ability on vascular smooth muscles. As a result, the presence of Anagliptin attenuated the senescent phenotype induced by IL-1β. Mechanistically, our knockdown experiment demonstrates that the action of Anagliptin is dependent on the activity of SIRT-1, implying that SIRT-1 is an essential element of the Anagliptin regulator upstream.

A recent study shows that selective elimination of senescent cells by silencing key regulators in mice can improve age-related chronic diseases [36]. It is optimistic that the targeted suppression of vascular cells could have the potential to slow down the progression of atherosclerosis. Previous works show the inhibition of the senescence pathway in vascular smooth muscle cells appears to affect atherosclerotic plaque vulnerability [37–39]. DPP-4 inhibitors have shown many beneficial effects in cardiovascular diseases [40,41]. It is possible that this class of drug could have a role in alleviating vascular smooth muscle cells senescence in hyperlipidemia conditions. Also, we have to mention the limitation of the current study. Although our study shows strong evidence that inhibition of the DPP4 pathway by Anagliptin could attenuate vascular smooth muscle cells senescence in cultured cells, the underlying mechanism remains incompletely understood. We show that SIRT-1 could be a central mediator of Anagliptin action, however, the detailed molecular pathways need to be investigated. Also, future *in vivo* experiments are warranted to validate the findings.

## Conclusion

To sum up, our data revealed that Anagliptin prevented IL-1β-induced cellular senescence in vascular smooth muscle cells by up-regulating SIRT1. Thus, we concluded that the DPP-4 inhibitor Anagliptin has a protective role in vascular smooth muscle cells.
